# Nonabsorbable Barbed Sutures for Diastasis Recti. A Useful Device with Unexpected Risk: Two Case Reports

**DOI:** 10.1055/a-2181-8382

**Published:** 2024-03-04

**Authors:** Lorenzo Giorgi, Veronica Ponti, Filippo Boriani, Andrea Margara

**Affiliations:** 1 Division of General Surgery, Department of Surgery, Humanitas S. Pio X Hospital, Milan, IT, Italy; 2 Department of Biomedical Sciences, Humanitas University, Pieve Emanuele, Milan, Italy; 3 Plastic Surgery Service, Humanitas S. Pio X Hospital, Milan, IT, Italy; 4 Department of Plastic Surgery and Microsurgery, University Hospital of Cagliari, University of Cagliari, Monserrato, CA, Italy

**Keywords:** diastasis recti, abdominal wall, nonabsorbable barbed suture, granulomas, polypropylene suture

## Abstract

The introduction of nonabsorbable barbed sutures in plastic surgery has allowed the achievement of significant results in terms of efficacy and short- and long-term outcomes. However, a nonabsorbable material with no antibacterial coating could act as a substrate for subclinical bacterial colonization and thereby determine recurrent subacute and chronic infective–inflammatory processes. The authors report a clinical experience of subacute infectious complications after two cases of diastasis recti surgical correction. The authors present a two-case series in which a nonabsorbable barbed suture was used for the repair of diastasis recti. The postoperative course was complicated by surgical site infection. The origin of the infectious process was clearly localized in the fascial suture used for diastasis correction. The suture was colonized by bacteria resulting in the formation of multiple granulomas of the abdominal wall a few months postoperatively. In both the reported cases, the patients partially responded to the antibiotic targeted therapy and reoperation was required. The microbiological analyses confirmed the colonization of sutures by *Staphylococcus aureus* . Barbed nonabsorbable sutures should be avoided for diastasis recti surgical correction to minimize the risk of infectious suture-related complications. The paper's main novel aspect is that this is the first clinical report describing infectious complications after surgical correction of diastasis recti with barbed polypropylene sutures. The risk of microbiological subclinical colonization of polypropylene suture untreated with antibacterial coating, therefore, should be taken into account.

## Introduction

During the last few decades, with the rising prevalence of obesity (worldwide obesity has nearly tripled since 1975) 1 and consequently of weight loss surgery, postbariatric body contouring plastic surgery has increased exponentially. Abdominoplasty, thigh lift, brachioplasty, mastopexy, upper and lower back lift are among the most performed postbariatric surgery procedures.

The growing interest in this field has resulted in the introduction of new suture materials and techniques to achieve the best results with the minimum incidence of complications. Barbed self-anchoring sutures are a relatively new type of surgical suture in which the multiple barbs anchor the tissue without knots, distributing tension evenly among the sutures, thus leading to a better wound healing while also reducing operative time. 2 In addition, many authors consider barbed sutures useful to diminish the skin fibrotic reaction as they dramatically diminish the number of knots and, therefore, the risk of granulomas.

Although some studies confirmed that barbed nonabsorbable sutures are not associated with an increased rate of adherence of bacteria according to in vitro wound models, 3 the in vivo actual risk remains unclear. 4

In this case series, we report two patients who underwent abdominoplasty and concurrent repair of diastasis recti by means of nonabsorbable undyed spiral barbed bidirectional polypropylene suture, calibre 2, named STRATAFIX, produced by ETHICON (Raritan, NJ). Both the cases were complicated by surgical site infection (SSI) in which the suture used for fascial repair was colonized by bacteria with formation of multiple granulomas on the abdominal wall along the midline, requiring reintervention in both the cases.

## Cases

## Case 1

In May 2017, a 37-year-old female, smoker, underwent abdominoplasty with diastasis recti correction following massive weight loss surgery (sleeve gastrectomy). The patient developed SSI with dehiscence of the surgical wound and formation of an abscess, which was drained and treated with broad-spectrum antibiotics and Negative Pressure Wound Therapy (NPWT). In July 2017, a CT scan showed the presence of fluid accumulation in the subcutaneous tissue (9 × 3 cm): the patient continued with NPWT until the wound was completely healed.

From this moment, the patient consulted numerous physicians in multiple hospitals in the area.

**Fig. 1:**
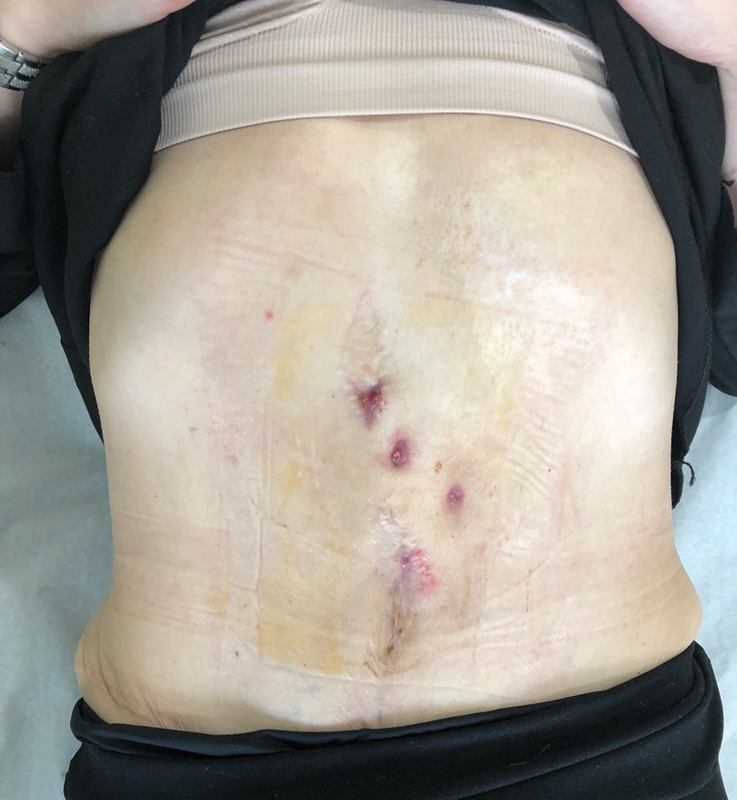
Skin ulcers appeared along the midline after 6 months from the abdominoplasty.

After 6 months, skin ulcers along the midline appeared and they were initially treated with antibacterial alginate and silver dressing ( Fig. 1 ). In February 2018, a wound culture from the ulcers tested positive for methicillin-resistant *Staphylococcus aureus* (MRSA) and adequate antibiotic therapy was started.

In May 2018, the patient accessed the Emergency Department (ED) upon referral from the attending physician in response to an abdominal CT scan that was compatible with an intra-abdominal abscess. During the physical examination, periumbilical wounds/fistulas were found, without pus. The liponecrosis induced by the partial resorption of the sutures utilized to treat the diastasis of recti muscles was hypothesized as the cause of the repeated abscess phenomena.

In July 2018, she was hospitalized due to progressive worsening of multiple pus-secreting fistulous tracts, for which the CT scan showed subcutaneous localization: in that period, she underwent surgery with complete abscess removal and NPWT. After 4 months of dressings and targeted antibiotic therapy, the wounds healed completely.

**Fig. 2:**
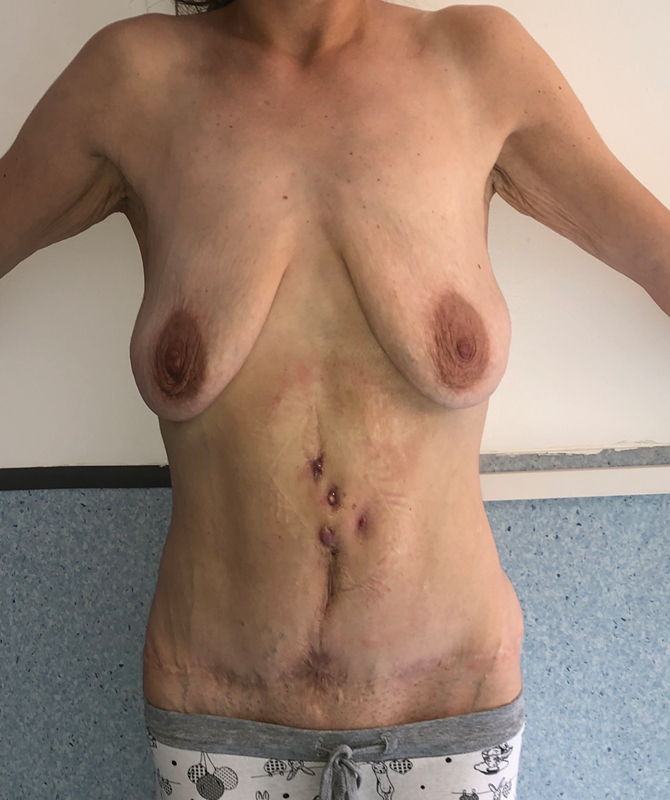
Reappearance of cutaneous lesions after umbilical hernia repair.

In August 2019, the patient was treated for umbilical hernia surgical repair in another clinic. The procedure was complicated by wound infection that was drained and treated with broad-spectrum antibiotics: the surgical report did not mention the placement of a mesh. After 3 months, the patient was admitted in the ED for multiple subcutaneous nodular formations, suspicious for abscesses, on the midline and at the periumbilical level ( Fig. 2 ). Blood chemistry tests were normal (negative inflammation indices) and abdominal ultrasound confirmed the presence of multiple fluid collections compatible with abscesses. A culture swab on the wound was performed and it tested positive for oxacillin and MRSA. The patient started targeted antibiotic therapy based on Teicoplanin, but showed slow and poor healing.

In May 2020, a surgical revision was performed in our center: on that occasion, the skin containing the ulcerations was removed and the fistulous tracts were followed deep to the superficial fascia of recti muscles; the nonabsorbable suture originally used for recti diastasis and an umbilical granuloma were completely removed. These findings were subjected to histological and culture examination which confirmed the presence of MRSA.

**Fig. 3:**
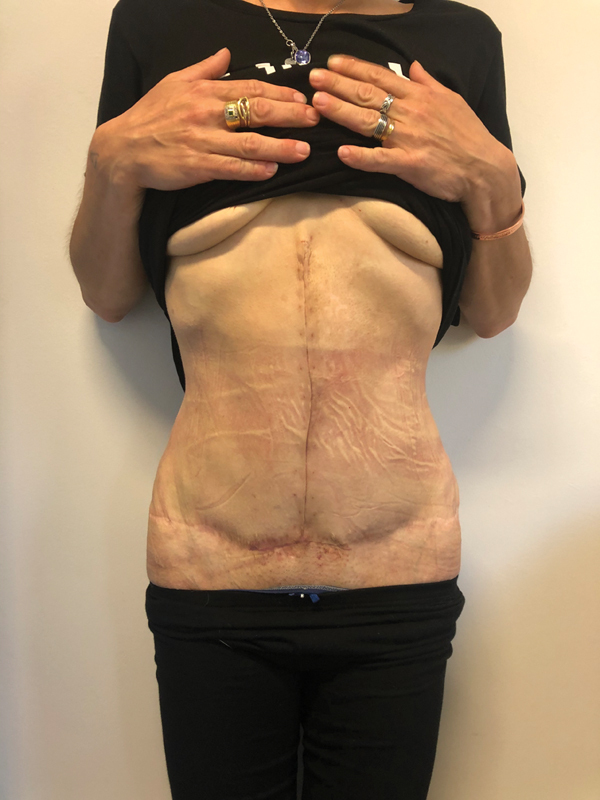
Final result.

The patient was then discharged and continued therapy at home, with scheduled follow-ups in our hospital ( Fig. 3 ).

## Case 2

The second patient was a 39-year-old female who in October 2019 underwent abdominoplasty with diastasis recti repair with nonabsorbable barbed bidirectional polypropylene suture after weight loss surgery (sleeve gastrectomy). The postoperative course was complicated by SSI in the middle third of the incision. The culture swab from the wound tested positive for *Staphylococcus aureus* (SA). The infection was promptly treated with targeted antibiotic therapy with complete healing of the wound within a few weeks.

**Fig. 4:**
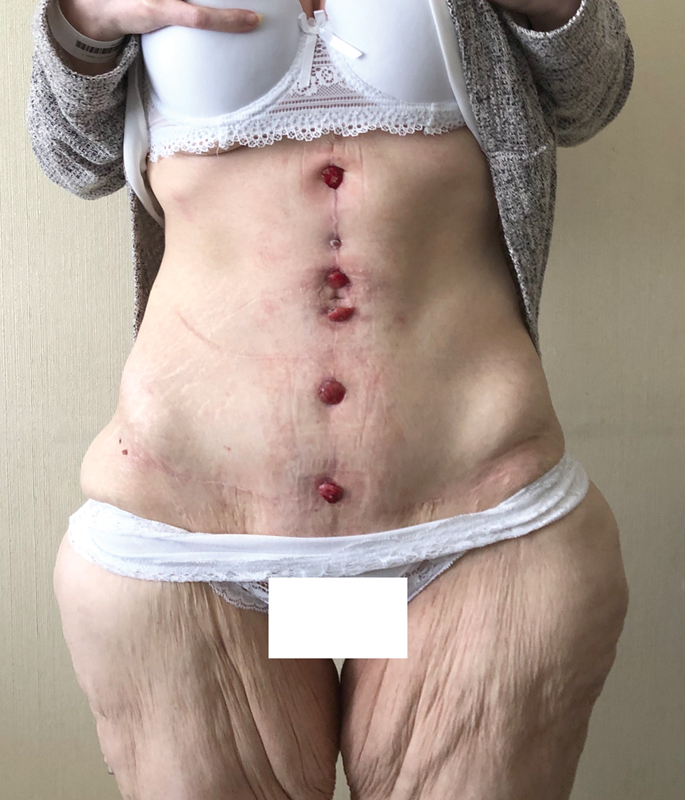
Multiple granulomas on the midline incision.

In the following course, small ulcerations appeared along the midline incision, progressively evolving into granulomatous nodules. These lesions were treated with multiple dressings but remained unsuccessful ( Fig. 4 ).

The patient finally underwent reintervention in May 2020, during which all the nodular lesions were incised and followed from the skin down to the recti fascia and the nonabsorbable suture used in the previous surgical intervention was completely removed. Interestingly, both the wound swab collected preoperatively and the suture tested positive for SA colonization.

The result after healing was optimal with complete patient satisfaction.

## Discussion

Diastasis recti is an anatomical condition in which the linea alba in the epigastrium appears to be thinner and presents as a midline bulging of the anterior abdominal wall. Surgical repair of diastasis recti can be performed simultaneously during an abdominoplasty or hernia repair and consists of either a single- or double-layer suture closure of the recti fascia, according to surgeon's preference. 5

There are currently no guidelines for the best suture to be used in recti diastasis repair 6 and even if traditionally the preferred suture materials for the fascia plication were nonabsorbable, new evidence suggests that long-acting absorbable sutures (barbed or smooth) are an effective alternative. 7

In these two cases, the recurrent and complicated ulcerations of the skin most probably had an origin from the surgical wound infection. Our hypothesis is that the nonabsorbable suture used for the synthesis of the fascia of recti muscles was colonized throughout its length by bacteria, in a sort of “ *subclinical infection* ” (as suggested by the fact that in both the patients, the culture confirmed the same pathogen—both superficially and on the suture once removed). The local and systemic antibiotic therapies were never capable of eradicating the infection, which led to recurrent phenomena of infectious exacerbation with abscesses and granulomas formation. The aggressive surgical therapy, with the complete removal of the granulomas, the fistulous tract, the infected skin, and the polypropylene suture seemed to be the only successful therapy capable of completely eliminating bacterial colonization.

The pathogenic role of the biofilm in determining chronic and subclinical bacterial colonization is supported by numerous recent studies 8 : biofilm-residing bacteria on nonabsorbable material can be resistant to both the immune system and antibiotics. 9 10 The current knowledge on how biofilm may contribute to the pathogenesis of disease indicates several different mechanisms: a reservoir of pathogenic bacteria that can trigger infectious acute/subacute manifestations, or even playing a more active role, for example, by contributing to chronic inflammation. A study by Chalya and coworkers 11 has shown in their 872 patients' series that nonabsorbable sutures, compared with absorbable sutures, are more frequently associated with stitch sinus and chronic pain. Rosen and Hartman 7 have reported in their series of 17 patients: two cases of minor seroma and one case of infected hematoma with long-acting barbed sutures made of polydioxanone. On the other hand, van Uchelen et al 12 observed with a cross-sectional study on patients treated with absorbable sutures ( *N* = 40) that 40% of them presented recurrent diastasis proven with ultrasound 6 months postoperatively.

Barbed sutures have the advantage of a reduced surgical time and consequently fewer indirect costs related to operation room, as described by Gutowski and Warner. 13

Antiseptic-coated sutures are seldom reported as a tool for correcting recti diastasis, but are increasingly employed for laparotomies. For correction of recti diastasis, the scientific evidence supporting antibiotic-coated sutures is little.

A multicenter randomized clinical trial including 1,224 patients explored rates of SSI in patients undergoing midline laparotomies. By comparing groups treated with uncoated PDS versus triclosan-coated suture (TCS) Polydioxanone (PDS) Plus for fascia closure, no differences were demonstrated in infection rates of the two groups. 14

Even though other studies have also failed to demonstrate any efficacy of antibiotic sutures in preventing SSIs, 15 proof that TCSs reduce the risk of surgical wound infection in all kinds of surgery has been described. 16 Table 1 reviews the types of coatings available in the medical literature and recent studies on TCSs.

**Table 1:** Coating types described in the medical literature

Coating type	Suture material	Availability in the market	Studies
Triclosan	Polyglactin Polydioxanone Poliglecapron	Yes	Onesti et al 2018 17
Metallic nanoparticles	Silk	No (in vitro studies)	Vieira et al. 2022 18
Silver nanoparticles	Silk Absorbable braided unspecified material	No (in vitro and in vivo studies)	Baygar et al 2019 19 Vorobyova et al 2022 20 Syukri et al 2020 21 Liu et al 2017 22
Propolis and biogenic silver nanoparticles	Silk	No (in vitro and in vivo studies)	Baygar 2020 23
Silver nanoparticles and hyperbranched polylysine	Polyglycolic acid	No (in vitro study)	Ho et al 2013 24
Zinc oxide nanoparticles	Gum	No (in vivo study)	Irfan et al 2022 25
Antimicrobial peptides	Spider silk	No (in vitro study)	Franco et al 2019 26
Berberine and Artemisinin	Silk	No (in vitro and in vivo studies)	Wang et al 2022 27
Triclosan (systematic review and meta-analysis)	Vicryl, Monocryl, PDS, Chinese silk	Yes (clinical studies)	Otto-Lambertz et al 2023 28
Triclosan (meta-analysis)	Vicryl	Yes (clinical studies)	He et al 2022 29
Triclosan and (clinical multicenter prospective trial and meta-analysis)	PDS, Vicryl	Yes (clinical study and meta-analysis)	Miyoshi et al 2022 30

Although the case series is short, based on this experience and on a careful review of the literature, the current evidence supports the recommendation to employ long-term absorbable sutures, to maintain a good degree of retention in the postoperative course, thereby allowing for the biologic timing necessary for collagen formation and remodeling, with effective abdominal wall continence. The evidence for recommending the use of coatings is weak and controversial.

### Conclusion

In conclusion, the permanent material used in these two cases (i.e., the polypropylene) can act as a substrate for the creation of bacterial biofilm, thus leading to a subacute/chronic inflammation resistant to multiple treatments. Our final suggestion in terms of suture choice for correction of diastasis is to prefer barbed long-acting absorbable sutures or sutures treated with some form of antibacterial coating, for example, TCS. 10

## References

[1] Apovian C M Obesity: definition, comorbidities, causes, and burden Am J Manag Care 2016 22 07 s176 s185 27356115

[2] Shermak M A The application of barbed sutures in body contouring surgery Aesthet Surg J 2013 33 03 72S 75S 24084881 10.1177/1090820X13499915

[3] Fowler J R Perkins T A Buttaro B A Truant A L Bacteria adhere less to barbed monofilament than braided sutures in a contaminated wound model Clin Orthop Relat Res 2013 471 02 665 671 23001503 10.1007/s11999-012-2593-z PMC3549181

[4] Chawla H van der List J P Fein N B Henry M W Pearle A D Barbed suture is associated with increased risk of wound infection after unicompartmental knee arthroplasty J Arthroplasty 2016 31 07 1561 1567 26872587 10.1016/j.arth.2016.01.007

[5] ElHawary H Abdelhamid K Meng F Janis J EA A comprehensive, evidence-based literature review of the surgical treatment of rectus diastasis Plast Reconstr Surg 2020 146 05 1151 1164 33136963 10.1097/PRS.0000000000007252

[6] Hernández-Granados P Henriksen N A Berrevoet F European Hernia Society guidelines on management of rectus diastasis Br J Surg 2021 108 10 1189 1191 34595502 10.1093/bjs/znab128 PMC10364860

[7] Rosen A Hartman T Repair of the midline fascial defect in abdominoplasty with long-acting barbed and smooth absorbable sutures Aesthet Surg J 2011 31 06 668 673 21813880 10.1177/1090820X11415242

[8] Vestby L K Grønseth T Simm R Nesse L L Bacterial biofilm and its role in the pathogenesis of disease Antibiotics (Basel) 2020 9 02 59 32028684 10.3390/antibiotics9020059 PMC7167820

[9] Kostakioti M Hadjifrangiskou M Hultgren S J Bacterial biofilms: development, dispersal, and therapeutic strategies in the dawn of the postantibiotic era Cold Spring Harb Perspect Med 2013 3 04 a010306 23545571 10.1101/cshperspect.a010306 PMC3683961

[10] Edmiston C E Seabrook G R Goheen M P Bacterial adherence to surgical sutures: can antibacterial-coated sutures reduce the risk of microbial contamination? J Am Coll Surg 2006 203 04 481 489 17000391 10.1016/j.jamcollsurg.2006.06.026

[11] Chalya P L Massinde A N Kihunrwa A Mabula J B Abdominal fascia closure following elective midline laparotomy: a surgical experience at a tertiary care hospital in Tanzania BMC Res Notes 2015 8 281 26121978 10.1186/s13104-015-1243-4 PMC4486392

[12] van Uchelen J H Kon M Werker P M The long-term durability of plication of the anterior rectus sheath assessed by ultrasonography Plast Reconstr Surg 2001 107 06 1578 1584 11335840 10.1097/00006534-200105000-00046

[13] Gutowski K A Warner J P Incorporating barbed sutures in abdominoplasty Aesthet Surg J 2013 33 03 76S 81S 24084882 10.1177/1090820X13499576

[14] Diener M K Knebel P Kieser M Effectiveness of triclosan-coated PDS Plus versus uncoated PDS II sutures for prevention of surgical site infection after abdominal wall closure: the randomised controlled PROUD trial Lancet 2014 384 9938 142 152 24718270 10.1016/S0140-6736(14)60238-5

[15] Canadian Agency for Drugs and Technologies in Health Rapid Response Reports. In:Antibacterial Sutures for Wound Closure After Surgery: A Review of Clinical and Cost-Effectiveness and Guidelines for Use Ottawa, ON: Canadian Agency for Drugs and Technologies in Health; 2014 25520998

[16] Konstantelias A A Andriakopoulou C S Mourgela S Triclosan-coated sutures for the prevention of surgical-site infections: a meta-analysis Acta Chir Belg 2017 117 03 137 148 28399780 10.1080/00015458.2017.1287396

[17] Onesti M G Carella S Scuderi N Effectiveness of antimicrobial-coated sutures for the prevention of surgical site infection: a review of the literature Eur Rev Med Pharmacol Sci 2018 22 17 5729 5739 30229851 10.26355/eurrev_201809_15841

[18] Vieira D Angel S N Honjol Y Engineering surgical stitches to prevent bacterial infection Sci Rep 2022 12 01 834 35039588 10.1038/s41598-022-04925-5 PMC8764053

[19] Baygar T Sarac N Ugur A Karaca I R Antimicrobial characteristics and biocompatibility of the surgical sutures coated with biosynthesized silver nanoparticles Bioorg Chem 2019 86 254 258 30716622 10.1016/j.bioorg.2018.12.034

[20] Vorobyova V Vasyliev G Uschapovskiy D Lyudmyla K Skiba M Green synthesis, characterization of silver nanoparticles for biomedical application and environmental remediation J Microbiol Methods 2022 193 106384 34826520 10.1016/j.mimet.2021.106384

[21] Syukri D M Nwabor O F Singh S Antibacterial-coated silk surgical sutures by ex situ deposition of silver nanoparticles synthesized with *Eucalyptus camaldulensis* eradicates infections J Microbiol Methods 2020 174 105955 32442657 10.1016/j.mimet.2020.105955

[22] Liu X Gao P Du J Zhao X Wong K KY Long-term anti-inflammatory efficacy in intestinal anastomosis in mice using silver nanoparticle-coated suture J Pediatr Surg 2017 52 12 2083 2087 28958713 10.1016/j.jpedsurg.2017.08.026

[23] Baygar T Characterization of silk sutures coated with propolis and biogenic silver nanoparticles (AgNPs); an eco-friendly solution with wound healing potential against surgical site infections (SSIs) Turk J Med Sci 2020 50 01 258 266 31655520 10.3906/sag-1906-48 PMC7080367

[24] Ho C H Odermatt E K Berndt I Tiller J C Long-term active antimicrobial coatings for surgical sutures based on silver nanoparticles and hyperbranched polylysine J Biomater Sci Polym Ed 2013 24 13 1589 1600 23574366 10.1080/09205063.2013.782803

[25] Irfan M Munir H Ismail H Characterization and fabrication of zinc oxide nanoparticles by gum *Acacia modesta* through green chemistry and impregnation on surgical sutures to boost up the wound healing process Int J Biol Macromol 2022 204 466 475 35157899 10.1016/j.ijbiomac.2022.02.043

[26] Franco A R Fernandes E M Rodrigues M T Antimicrobial coating of spider silk to prevent bacterial attachment on silk surgical sutures Acta Biomater 2019 99 236 246 31505301 10.1016/j.actbio.2019.09.004

[27] Wang X Liu P Wu Q Sustainable antibacterial and anti-inflammatory silk suture with surface modification of combined-therapy drugs for surgical site infection ACS Appl Mater Interfaces 2022 14 09 11177 11191 35192338 10.1021/acsami.2c00106

[28] Otto-Lambertz C Decker L Adams A Yagdiran A Eysel P Can triclosan-coated sutures reduce the postoperative rate of wound infection? Data from a systematic review and meta-analysis Surgery 2023 174 03 638 646 37328397 10.1016/j.surg.2023.04.015

[29] He P Liu Z Chen H Huang G Mao W Li A The role of triclosan-coated suture in preventing surgical infection: a meta-analysis Jt Dis Relat Surg 2023 34 01 42 49 36700262 10.52312/jdrs.2023.842 PMC9903111

[30] Clinical Study Group of Osaka University, Colorectal Cancer Treatment Group (CSGOCG) Miyoshi N Fujino S Nishimura J Effectiveness of triclosan-coated sutures compared with uncoated sutures in preventing surgical site infection after abdominal wall closure in open/laparoscopic colorectal surgery J Am Coll Surg 2022 234 06 1147 1159Erratum in: J Am Coll Surg. 2022 Dec 1;235(6):968–969. Erratum in: J Am Coll Surg. 2022 Oct 1;235(4):689–69135703813

